# Supplementary material for: MAP1B mutations cause intellectual disability and extensive white matter deficit
Source: Nat Commun. 2018 Aug 27;9:3456. doi: 10.1038/s41467-018-05595-6 (PMC6110722; doi:10.1038/s41467-018-05595-6)
Supplement: Supplementary file 1 — Supplementary Information [file 41467_2018_5595_MOESM1_ESM.pdf]

MAP1B mutations cause intellectual disability and extensive white matter deficit

Walters GB et al.

SUPPLEMENTARY INFORMATION

Supplementary Table 1. MAP1B LoF carrier status, neuropsychiatric traits and intellectual functioning.
Supplementary Table 2. Head circumference, height and weight comparison for MAP1B LoF carriers (n = 9) compared with controls (n = 2,200).
Supplementary Table 3. MAP1B coding variants previously reported in literature.
Supplementary Table 4. MAP1B coding variants reported in public databases.
Supplementary Table 5. MAP1B copy number variations reported in public databases.

Supplementary Figure 1. Schematic of MAP1B exons and MAP1B protein (canonical transcripts).
Supplementary Figure 2. Pedigree plots of MAP1B families indicating the most likely carrier of the de novo event.
Footnote for Supplementary Figure 2.
Supplementary Figure 3. Plot of mean IQ and cognitive assessment scores in MAP1B LoF carriers compared with controls.
Footnote for Supplementary Figure 3.
Supplementary Figure 4. Plot of IQ versus polygenic score for IQ (PGS-IQ) in MAP1B carriers compared with controls.
Supplementary Figure 5. Plot of IQ versus polygenic score for educational attainment (PGS-EDU) in MAP1B carriers compared with controls.
Supplementary Figure 6. Corpus callosum and brain-wide white matter volume averages.
Supplementary Figure 7. Greyscale images of MAP1B antibody binding on a western blot.

Supplementary Data 1. Effects on structural MRI brain volumes, surface areas and thickness in MAP1B LoF carriers compared with controls. Separate file.

Supplementary References.

SUPPLEMENTARY TABLES

Supplementary Table 1. *MAP1B* LoF carrier status, neuropsychiatric traits and intellectual functioning.

Family #-Alias (Gender)[a]	Variant carrier status (mode of inheritance)[b]	Clinical features and functioning[c]	WASI[IS] / WISC (III/IV) / WPPSI-R (Methods)[d]	PVNH[e]
FAM1-A1 (F)	E712KfsTer10 (*De novo* - paternal background)	Alzheimers, Education (below average)		-
FAM1-B1 (F)	E712KfsTer10 (maternally inherited)	ID, Hyperactive, OCD, Education (below average)	WASI[IS]: FSIQ56, VIQ59, PIQ59	yes
FAM1-B2 (-)	Noncarrier			-
FAM1-B3 (M)	Noncarrier	Education (average), Mild Dyslexia, ADHD symptoms	WASI[IS]: FSIQ71, VIQ73, PIQ75	no
FAM1-C1 (F)	E712KfsTer10 (maternally inherited)	ID, Dyslexia, Dyscalculia	WASI[IS]: FSIQ56, VIQ62, PIQ55	yes
FAM1-C2 (-)	E712KfsTer10 (maternally inherited)	ID, ADD, OCD, Dyslexia	WASI[IS]: FSIQ63, VIQ61, PIQ70	-
FAM1-C3 (-)	Noncarrier			-
FAM1-C4 (M)	Noncarrier			-
FAM1-D1 (-)	E712KfsTer10 (maternally inherited)	ID, Dyslexia	WASI[IS]: FSIQ66, VIQ73, PIQ63<br>WISC-III: FSIQ62, VIQ64, PIQ64	yes
FAM1-D2 (-)	E712KfsTer10 (maternally inherited)	ID, ADHD (impulsive), NVLD, disorder of scholastic skills, behavioral and emotional disorder	WASI[IS]: FSIQ71, VIQ82, PIQ62<br>WISC-IV: FSIQ58, VC70, PR51, WM64, PS79	yes
FAM1-D3 (-)	E712KfsTer10 (maternally inherited)	ID, Dyslexia, Dyscalculia, Epilepsy	WASI[IS]: FSIQ58, VIQ62, PIQ59<br>WISC-III: FSIQ61, VIQ66, PIQ60	yes
FAM1-D4 (-)	Noncarrier	ID, ASD symptoms (not specified), ADD, disorder of scholastic skills (reading relatively spared), disorder of conduct and emotions	WISC-IV: FSIQ57, VC68, PR68, WM51, PS79	no
FAM1-D5 (-)	NA		WPPSI-R: FSIQ72, VIQ61, PIQ89	-
FAM1-E1 (-)	E712KfsTer10 (maternally inherited)	ID, behavioral problems and language delay	WPPSI-R: FSIQ66, VIQ73, PIQ65	-
FAM1-X1, X2, X3 (-)	Noncarrier			-
FAM2-G1 (M)	Noncarrier			no
FAM2-G2 (F)	Noncarrier	Education (below average), Migraine		-
FAM2-H1 (F)	E1032Ter (*De novo* - paternal background)	Dyslexia, Dyscalculia	WASI[IS]: FSIQ77, VIQ89, PIQ67	not discernable
FAM2-H2 (M)	Noncarrier		WASI[IS]: FSIQ87, VIQ86, PIQ90	no
FAM2-J1 (-)	E1032Ter (maternally inherited)	ID, ASD (ICD10-F84.1), ADHD (ICD10-F90.0), disorder of motor function, NVLD, disorder of scholastic skill	WISC-IV: FSIQ69, VC96, PR65, WM67, PS73	yes
FAM2-J2 (-)	E1032Ter (maternally inherited)	Dyslexia, Dyscalculia	WASI[IS]: FSIQ79, VIQ80, PIQ82	yes
FAM2-J3 (-)	E1032Ter (maternally inherited)	ASD (ICD10-F84.1), ID, ADHD, Late talker, NVLD	WISC-IV: FSIQ67, VC90, PR65, WM51, PS89	-
FAM2-J4 (-)	Noncarrier			-
FAM2-J5 (-)	Noncarrier			-
FAM2-X4, X5 (-)	Noncarrier			-
FAM3-K1 (M)	Noncarrier		WASI[IS]: FSIQ98, VIQ103, PIQ93	no
FAM3-K2 (F)	Noncarrier	Education (below average)	WASI[IS]: FSIQ103, VIQ103, PIQ101	no
FAM3-L1 (F)	R1664Ter (*De novo* - unknown)		WASI[IS]: FSIQ92, VIQ105, PIQ82	yes
FAM3-L2 (-)	Noncarrier			-
FAM3-L3 (M)	NA			-
FAM3-M1 (F)	R1664Ter (maternally inherited)	ASD (ICD10-F84.5), disorder of scholastic skill	WISC-IV: FSIQ76, VC94, PR93, WM63, PS75	-
FAM3-M2 (-)	Noncarrier			-
FAM4 (M)	5q11.2-13.2 deletion (14.7 MB) (*De novo* - paternal background)	ID (severe)		-

[a]FAM refers to family, F for female, M for male and – for gender not reported here.
[b]GenBank: NP_005900[https://www.ncbi.nlm.nih.gov/protein/153945728] (GenBank: NM_005909[https://www.ncbi.nlm.nih.gov/nuccore/NM_005909.4], hg38): E712KfsTer10 (c.2133delG, chr5:72195488 (G/-)), E1032Ter (c.3094G>T, chr5:72196449 (G/T)), R1664Ter (c.4990C>T, chr5:72198345 (C/T)).
[c]Education is year of birth and gender adjusted number of years in education system (average is defined as ± 0.5 SD). ADD, Attention deficit disorder; ADHD, Attention deficit/Hyperactivity disorder; ASD, Autism spectrum disorder; ID, Intellectual disability; NVLD, Nonverbal learning disorder; OCD, Obsessive compulsive disorder.
[d]WASI[IS] - Wechsler Abbreviated Scale of Intelligence (Icelandic version)[1,2], WPPSI-R - Wechsler Preschool and Primary Scale of Intelligence[3], WISC - Wechsler Intelligence Scale for Children (III and IV)[4,5], IQ – Intelligence Quotient, VIQ – Verbal IQ, PIQ – Performance IQ, FSIQ – Full Scale IQ, VC - Verbal Comprehension, PR – Perceptual Reasoning, WM – Working Memory, PS – Processing Speed.
[e]PVNH – Periventricular nodular heterotopia, clinical magnetic resonance imaging (MRI) evaluation.

Supplementary Table 2. Head circumference, height and weight for *MAP1B* LoF carriers (n = 9) compared with controls (n = 2,200).

Trait	*MAP1B* mean (SD)	Control mean (SD)	β	*P*
Head circumference (cm)	55.5 (1.3)	57.4 (2.3)	-0.62	0.16
Height (m)	1.7 (0.2)	1.7 (0.1)	-0.011	0.98
Weight (kg)	86.8 (17.8)	82.9 (21.4)	0.26	0.56

The mean (SD) values are unadjusted measurements of head circumference (in centimeters), height (in meters) and weight (in kilograms). For analyses the measurements were inverse normal transformed followed by adjusted for sex, age and age^2. Values were then shifted and scaled, resulting in controls having a mean of 0 and a standard deviation of 1. The effects (β in SD) and *P-values* were calculated by comparing *MAP1B* LoF carriers with controls using a generalised least squares regression with a variance-covariance matrix based on the kinship coefficient of each pair of individuals. The β represents the effect of the *MAP1B* LoF carrier status on the three traits.

Dysmorphic features, based on Tripi et al.[6] were also evaluated by the interviewing psychologist, including head shape/cephalic index, prominent forehead, micrognathia, inner-canthal distance (hyper–hypotelorism), fused eyebrows, epicanthus, ptosis, low set ears or ear anomalies, philtrum length, thin upper lip, cleft lip or palate (uvula), finger length, and finger or nail anomalies. None of the features were in excess in the *MAP1B* LoF carriers.

Supplementary Table 3. *MAP1B* coding variants previously reported in literature.

Literature Source	hg38	hg19	Alleles	Protein	Comment
Iossifov et al. 2012, Ref. [7]	72195128	71490955	C/A	D591E	de novo missense in a female with ASD (quadid 12220)
Iossifov et al. 2014, Ref. [8]	72199099	71494926	C/T	S1915F	missense transmitted from father to unaffected daughter who is a sibling of an individual with ASD (family id 14637)
De Rubeis et al. 2014, Ref. [9]	72195267	71491094	G/T	V638L	missense in a female with ASD (Autism Sequencing Consortium-ASC, Child_ID:09C81948)
Krumm et al. 2015, Ref. [10]	72107534	71403361	G/A	M1I	start lost missense transmitted from father to son with Asperger (family id 13383)
Krumm et al. 2015, Ref. [10]	72107534	71403361	G/A	M1I	start lost missense transmitted from father to unaffected son who is a sibling of a male with Autism (family id 13534)
Krumm et al. 2015, Ref. [10]	72194265	71490092	G/A	V304M	missense transmitted from father to unaffected son who is a sibling of a male with Autism (family id 13984)
Krumm et al. 2015, Ref. [10]	72194265	71490092	G/A	V304M	missense transmitted from mother to son with PDD and unaffected daughter (family id 14664)
Krumm et al. 2015, Ref. [10]	72197922	71493749	G/A	E1523K	missense transmitted from father to son with PDD (family id 12802)
Krumm et al. 2015, Ref. [10]	72197922	71493749	G/A	E1523K	missense transmitted from mother to son with Autism and unaffected daughter (family id 12833)
Krumm et al. 2015, Ref. [10]	72205237	71501064	T/A	Ter2469K	Stop lost missense transmitted from mother to unaffected son who is a sibling of a female with Autism (family id 13335)
DDD (McRae et al.) 2017, Ref. [11]	72195326	71491153	G/-	K657KfsTer23	*de novo* frameshift LoF in a male with DD (DDD4K.01352)
DDD (McRae et al.) 2017, Ref. [11]	72200116	71495943	C/T	S2254L	missense in a male with DD (DDD4K.01526)
Kosmicki et al. 2017, Ref. [12]	72194697	71490524	A/T	K448Ter	stop gain in a male control (nn.4402-S3)
Simons Simplex Collection[a]	72107559	71403386	G/T	E10Ter	stop gain in father of a male with ASD (TO2_FB12121FA)
Simons Simplex Collection[a]	72199287	71495114	G/T	E1978Ter	stop gain in mother of a female with ASD (TO2_14444MO)

[a]Simons Simplex Collection (SSC) data was accessed through the WuXi NextCODE-SSC portal [https://simons.wuxinextcode.com/csa/projects/10025/dashboard]. We are grateful to all of the families at the participating SSC sites, as well as the principal investigators (A. Beaudet, R. Bernier, J. Constantino, E. Cook, E. Fombonne, D. Geschwind, R. Goin-Kochel, E. Hanson, D. Grice, A. Klin, D. Ledbetter, C. Lord, C. Martin, D. Martin, R. Maxim, J. Miles, O. Ousley, K. Pelphrey, B. Peterson, J. Piggot, C. Saulnier, M. State, W. Stone, J. Sutcliffe, C. Walsh, Z. Warren, E. Wijsman). We appreciate obtaining access to phenotypic and genotypic data on SFARI Base via the WuXi NextCODE-SSC portal. Approved researchers can obtain the SSC population dataset referred to in the above table by applying at https://base.sfari.org/, as well as access to the WuXi NextCODE-SSC portal, which hosts the data in a cloud environment with integrated analytical tools.

The following query string was used to identify the two variants, in the Simons Simplex Collection (SSC) data through the WuXi NextCODE-SSC portal:

```
def ##ref## = ref;
 def ##vep_single## = source/anno/vep_v85/vep_single_wgs.gord;
 def ##freqmax## = ##ref##/freq_max.gorz | select 1-4,max_af;
 def ##VEPfreq## = ##vep_single## | where max_consequence in
('transcript_ablation','splice_acceptor_variant','splice_donor_variant','stop_gained','frameshift_variant','stop_lost','start_lost','transcript_amplification') | varjoin -r -l -e 0.0 <(gor ##freqmax##);
gor #genes# | where gene_symbol in ('MAP1B') | join -segvar <(gor ##VEPfreq##) -xr gene_symbol -xl gene_symbol | where max_consequence in
('transcript_ablation','splice_acceptor_variant','splice_donor_variant','stop_gained','frameshift_variant','stop_lost','start_lost','transcript_amplification') | where isfloat(max_Af) and float(max_Af) <= 0.05 | select
1,Pos- | rename Gene_symbolx Gene_Symbol | sort 3000000 | varjoin -r <(gor #wgsvars# -f 'TO2_14692FA',....ALL_SUBJECT_IDs....,'TO2_11025FA' ) | where Depth = 9999 or GL_Call >= 5 and Depth >= 8 and
(CallCopies = 2 and CallRatio >= 0.66 or CallCopies = 1 and CallRatio >= 0.2 and CallRatio <= 1.0-0.2) |group 1 -gc
Reference,Call,Max_Impact,max_consequence,Biotype,Gene_Symbol,Transcript_count,Amino_Acids,Protein_Position,CDS_position,Refgene,MAX_AF -count -sc PN -lis -len 20000|rename allCount PN_count
```

Supplementary Table 4. *MAP1B* coding variants reported in public databases.

Database Source[a]	hg38	hg19	Alleles	Protein	rs-ID	ExAc/gnomAD allele frequency[b]	Carriers[c]
gnomAD	72115745	71411572	G/T	E78Ter		- / 0.0000323	one African
ExAc/gnomAD	72186641	71482468	C/T	R133Ter	rs773674867	0.000008237 / 0.000004062	one European (Non-Finnish)
ExAc/gnomAD	72186650	71482477	-/T	L137AfsTer9	rs753828375	0.000008237 / 0.000004061	one European (Non-Finnish)
ExAc/gnomAD/EVS-ESP	72194250	71490077	C/T	R299Ter	rs146324682	- / 0.00003229	one European (Non-Finnish)
ExAc/gnomAD	72194697	71490524	A/T	K448Ter	rs770530337	0.000008242 / 0.000004062	one European (Non-Finnish)
dbSNP	72194811	71490638	C/T	R486Ter	rs868169901		Not available
ExAc	72194889	71490716	A/-	K512SfsTer19	rs760995541	0.000008353 / -	one European (Non-Finnish)
dbSNP	72194908	71490735	A/-	Q518QfsTer12	rs36100729		Not available
ExAc	72195092	71490919	GTTGAAAGCAAAG/-	V580KfsTer3		0.000008307 / -	one East Asian
ExAc	72195101	71490928	C/-	S582RfsTer5	rs765350019	0.00002497 / -	two African
EVS-ESP	72195224	71491051	GC/-	Q624SfsTer23			two European American
DECIPHER	72195326	71491153	G/-	K657KfsTer23			one heterozygous with multiple abnormalities
EVS-ESP	72195391	71491218	-/C	E679DfsTer23	rs896243481		ten European American and 20 African American
dbSNP	72195405	71491232	G/-	V684SfsTer37	rs867533822		Not available
EVS-ESP	72195414	71491241	-/CCTGA	E687AfsTer2	rs903414821		12 European American and 23 African American (plus one homozygous AA)
EVS-ESP	72195418	71491245	T/-	I688TfsTer34			ten European American and 24 African American (plus one homozygous AA)
gnomAD	72195507	71491334	A/T	K718Ter		- / 0.000004616	one Latino
ExAc/1000Genomes	72197175	71493002	G/T	E1274Ter	rs186784968	0.000008241 / -	one European (Non-Finnish)
ExAc	72199249	71495076	-/C	E1968RfsTer8	rs775893760	0.00002493 / -	three European (Non-Finnish)
ExAc/gnomAD/1000Genomes	72199526	71495353	T/G	Y2057Ter	rs548994956	0.000008247 / 0.00002031	four Latino and one Other
gnomAD	72199714	71495541	A/-	K2121SfsTer115		- / 0.000004063	one South Asian

[a]1000Genomes = 1000 Genomes Project (http://www.1000genomes.org/); dbSNP = Database of single nucleotide polymorphisms (https://www.ncbi.nlm.nih.gov/projects/SNP/snp_ref.cgi?geneId=4131); DECIPHER = DatabasE of genomiC variation and Phenotype in Humans using Ensembl Resources v9.19 (https://decipher.sanger.ac.uk/); EVS-ESP = Exome Sequencing Project-Exome Variant Server (http://evs.gs.washington.edu/EVS/); ExAc = Exome Aggregation Consortium (http://exac.broadinstitute.org/gene/ENSG00000131711); gnomAD = Genome Aggregation Database (http://gnomad.broadinstitute.org/gene/ENSG00000131711). Only variants observed in the canonical transcript included; ENSEMBL: ENST00000296755-ENSP00000296755 [https://www.ensembl.org/Homo_sapiens/Transcript/Summary?db=core;g=ENSG00000131711;r=5:72107234-72209570;t=ENST00000296755], RefSeq: NM_005909[https://www.ncbi.nlm.nih.gov/nuccore/NM_005909.4]-NP_005900[https://www.ncbi.nlm.nih.gov/protein/153945728]

[b]Only variants with a PASS filter in the ExAc or gnomAD databases were included. *MAP1B* LoF variation carrier frequency in both ExAc and gnomAD is estimated to be around 1/10,000.

[c]Heterozygous carriers unless otherwise stated.

Supplementary Table 5. *MAP1B* copy number variations reported in literature or public databases.

Source[a]	Source ID	Size	Inheritance	Phenotype(s)	Reference
DECIPHER	257500	2.41 Mb triplication	Unknown	Unknown	13
DECIPHER	265175	3.84 Mb duplication	De novo constitutive	Intellectual disability, Macrocephaly	13
DECIPHER	270911	7.18 Mb duplication	Unknown	Autistic behavior, Clinodactyly of the 5th finger, Delayed speech and language development, Global developmental delay, Intellectual disability, Overlapping toe	13
DECIPHER	273739	754.04 kb duplication	Inherited from parent with unknown phenotype	Unknown	13
DECIPHER	303204	208.20 kb deletion	Paternally inherited, constitutive in father	Abnormality of the face	13
Liu et al. 2015	10–17471	966 kb deletion	Denovo	Intellectual disability, seizures, borderline microcephaly, normal cranial MRI	14
VarView -NCBI	nssv1602328	74.39 Mb duplication		Developmental delay AND/OR other significant developmental or morphological phenotypes	15
VarView -NCBI	nssv13638981	181.18 Mb duplication		Abnormal facial shape; Intrauterine growth retardation; Micrognathia; Syndactyly; Ventricular septal defect	15
VarView -NCBI	nssv13655232	181.18 Mb duplication		Abnormality of the ear; Polydactyly; Short stature; obsolete Malformation of the heart and great vessels	15
VarView -NCBI	nssv13640215	181.20 Mb duplication		Global developmental delay	15
VarView -NCBI	nssv13648398	159.52 Mb duplication		Camptodactyly; Cleft palate; Dolichocephaly; Feeding difficulties; Microglossia; Pierre-Robin sequence; Respiratory distress	15
VarView -NCBI	nssv3396043	647.024 kb duplication		Developmental delay AND/OR other significant developmental or morphological phenotypes	15
VarView -NCBI	nssv466211	62.05 Mb duplication			16
VarView -NCBI	nssv466212	131.11 Mb deletion			16
VarView -NCBI	essv6984884	94.19 Mb duplication			17
VarView -NCBI	nssv1161363	16.97 Mb duplication			18
VarView -NCBI	nssv1161365	1.04 Mb duplication			18
VarView -NCBI	nssv3459625	16.97 Mb duplication			19
VarView -NCBI	nssv3446828	1.04 Mb duplication			19

[a]Database or Publication:DECIPHER = DatabasE of genomiC variation and Phenotype in Humans using Ensembl Resources v9.19 (https://decipher.sanger.ac.uk/); VarView-NCBI = Variation Viewer-NCBI (https://www.ncbi.nlm.nih.gov/variation/view/).

SUPPLEMENTARY FIGURES

Supplementary Figure 1. Schematic of *MAP1B* exons and MAP1B protein (canonical transcripts). a) Position of LoF variants in exons to scale (introns are not to scale) and **b)** relative to protein domains**.** All of the LoF variants are located in exon 5 (hg38:chr5:72193866-72200367) of *MAP1B*. No other LoF variants were found in *MAP1B* in the Icelandic sample. The *MAP1B* amino acid positions are in GenBank: NP_005900[https://www.ncbi.nlm.nih.gov/protein/153945728] and c. (coding) positions are in cDNA GenBank: NM_005909[https://www.ncbi.nlm.nih.gov/nuccore/NM_005909.4]. E712KfsTer10 (c.2133delG, hg38:chr5:72195488 (G/-)), E1032Ter (c.3094G>T, hg38:chr5:72196449 (G/T)), R1664Ter (c.4990C>T, hg38:chr5:72198345 (C/T)). E – Exons, UTR – Untranslated region, HC – Heavy chain, LC1 – Light chain 1, MTB - microtubule-binding domain, AB - actin-binding domain, MTA - microtubule assembly helping site, putative. See Entrez for *MAP1B*[https://www.ncbi.nlm.nih.gov/gene/4131] gene information. The red dotted lines represent the ExAc variants listed in Supplementary Table 4.

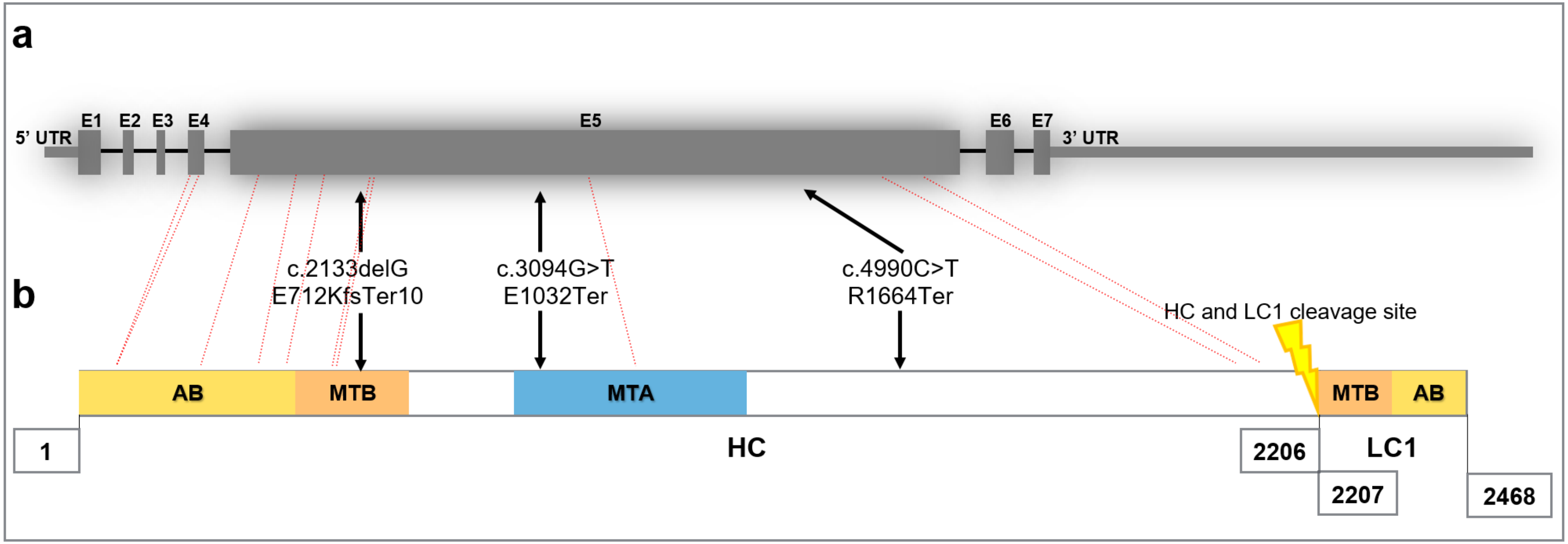

Supplementary Figure 2. Pedigree plots of *MAP1B* families indicating the most likely carrier of the *de novo* event. a) Family 1 (FAM1). The c.2133delG variant (E712KfsTer10) was transmitted from the great-grandmother (FAM1-A1) to the grandmother (FAM1-B1), and into two of her children (FAM1-C2 and FAM1-C1). Then from FAM1-C1 to the index cases (FAM1-D1-D2-D3) and one of their offspring (FAM1-E1). Parent-of-origin data indicate that FAM1-A1 inherited the chromosome carrying the variation from her father. Sanger sequencing three FAM1-A1's siblings' descendants (X1, X2, and X3), who shared the paternal haplotype harboring the variant, found that none carried the c.2133delG variant. Therefore, a *de novo* mutation most likely occurred during gametogenesis in FAM1-A1's father. **b)** Family 2 (FAM2). The siblings (FAM2-J1-J2-J3) inherited the c.3094G>T variant (E1032Ter) from their mother (FAM2-H1) and parent-of-origin data indicated that it was transmitted from her father (FAM2-G1) who in turn inherited the chromosome carrying the variation from his mother. We were unable to sequence either FAM2-G1 or his mother. However, we sequenced two of FAM2-G1's siblings (X4, X5) who shared the haplotype harboring the variant, but neither of them carried the c.3094G>T variant. Therefore, the mutation most likely occurred during gametogenesis in FAM2-G1 since it is unlikely to have occurred in his mother as out of her three children, all share the same haplotype from her, only FAM2-G1 has the variation. **c)** Family 3 (FAM3). According to parent-of-origin data, FAM3-L1 and her sibling (FAM3-L2) carried the same haplotypes from their parents at the *MAP1B* locus. However, Sanger sequencing revealed that neither the sibling nor either parent carried the c.4990C>T variant (R1664Ter). Hence, the *de novo* mutation originated in one of the parents (FAM3-K1 or FAM3-K2).

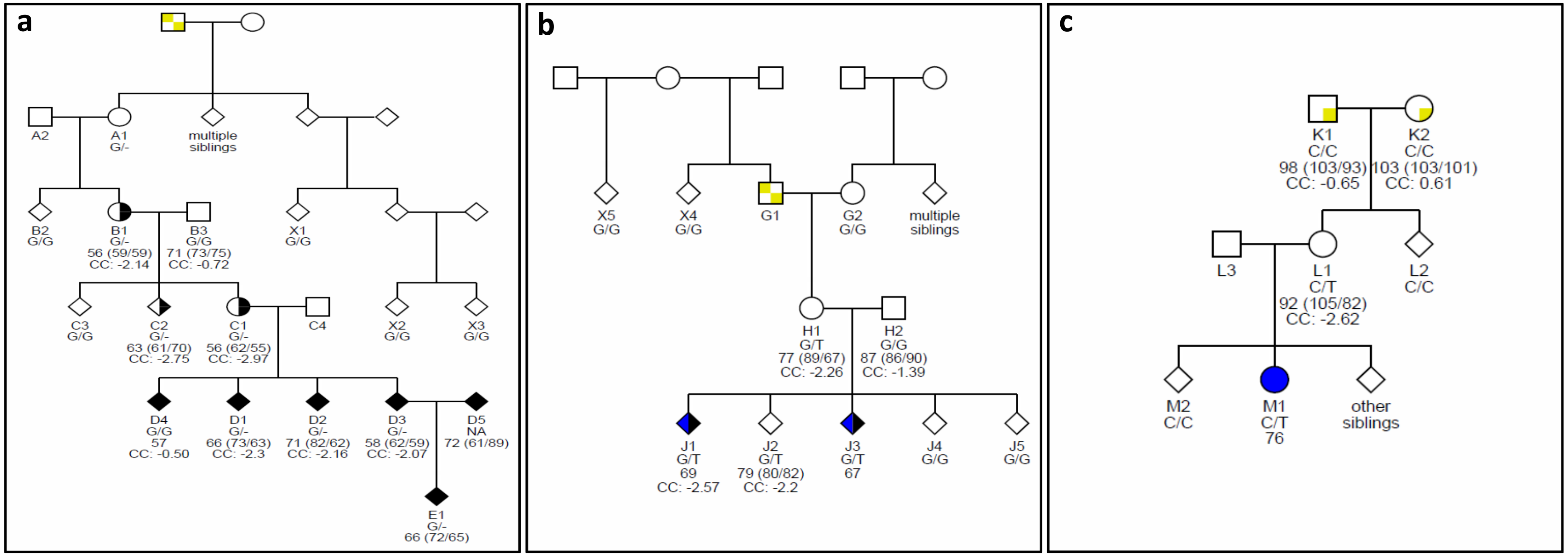

Footnote for Supplementary Figure 2. Squares are male, circles are female, diamonds are where gender is withheld, unfilled are unaffected, filled with black are intellectual disability affected, half black and white are individuals without a clinical diagnosis but an IQ below 70, blue are Autism spectrum disorder affected and yellow and white squares within the pedigree symbol indicates where it is most likely the initial *MAP1B* LoF mutation event occurred. Below each icon is the subjects: alias; *MAP1B* LoF variant genotype; Full Scale IQ (Verbal IQ / Performance IQ) from WASI[IS] or WPPSI-R (FAM1-B1,-B3,-C1,-C2,-D1,-D2,-D3; FAM2-H1,-H2,-J2; FAM3-K1,-K2,-L1 or FAM1-D5,-E1, respectively), or only Full Scale IQ reported from WISC-IV (FAM1-D4, FAM2-J1,-J3 and FAM3-M1), and Corpus callosum (CC) volume (all individuals with structural MRI also have DTI except FAM1-C1).

Supplementary Figure 3. Plot of mean IQ and cognitive assessment scores in *MAP1B* LoF carriers compared with controls. a) IQ (WASI[IS], WISC-IV or WPPSI-R) and WASI[IS] subtests, **b)** cognitive test battery scores. *MAP1B* LoF carriers (n = 13 for FSIQ and n = 9 for subtests; FAM1-B1,-C1,-C2,-D1,-D2,-D3,-E1, FAM2-H1,-J1,-J2,-J3, FAM3-L1,-M1) and controls (n = 2226 for IQ and n = 1768 for subtests; see Tables 1 and 2 for the number of cognitively assessed individuals in each group). IQ and cognitive test scores were inverse normal transformed. Cognitive test scores were also adjusted for sex, age and age^2. Note, adjustment for relatedness is not applied, as in Tables 1 and 2. IQ and cognitive test scores were then shifted and scaled so that controls had a mean of 0 and a standard deviation of one. While the lower IQ scores represent greater impairment, the cognitive test scores were arranged such that higher scores indicate greater impairment, in *MAP1B* LoF carriers. Bars either side of mean values represent 95% confidence interval (95 % CI). Dotted grey line in **(b)** is the average score (0.92 SD), in the *MAP1B* LoF carriers, for all cognitive tasks (excluding GAF).

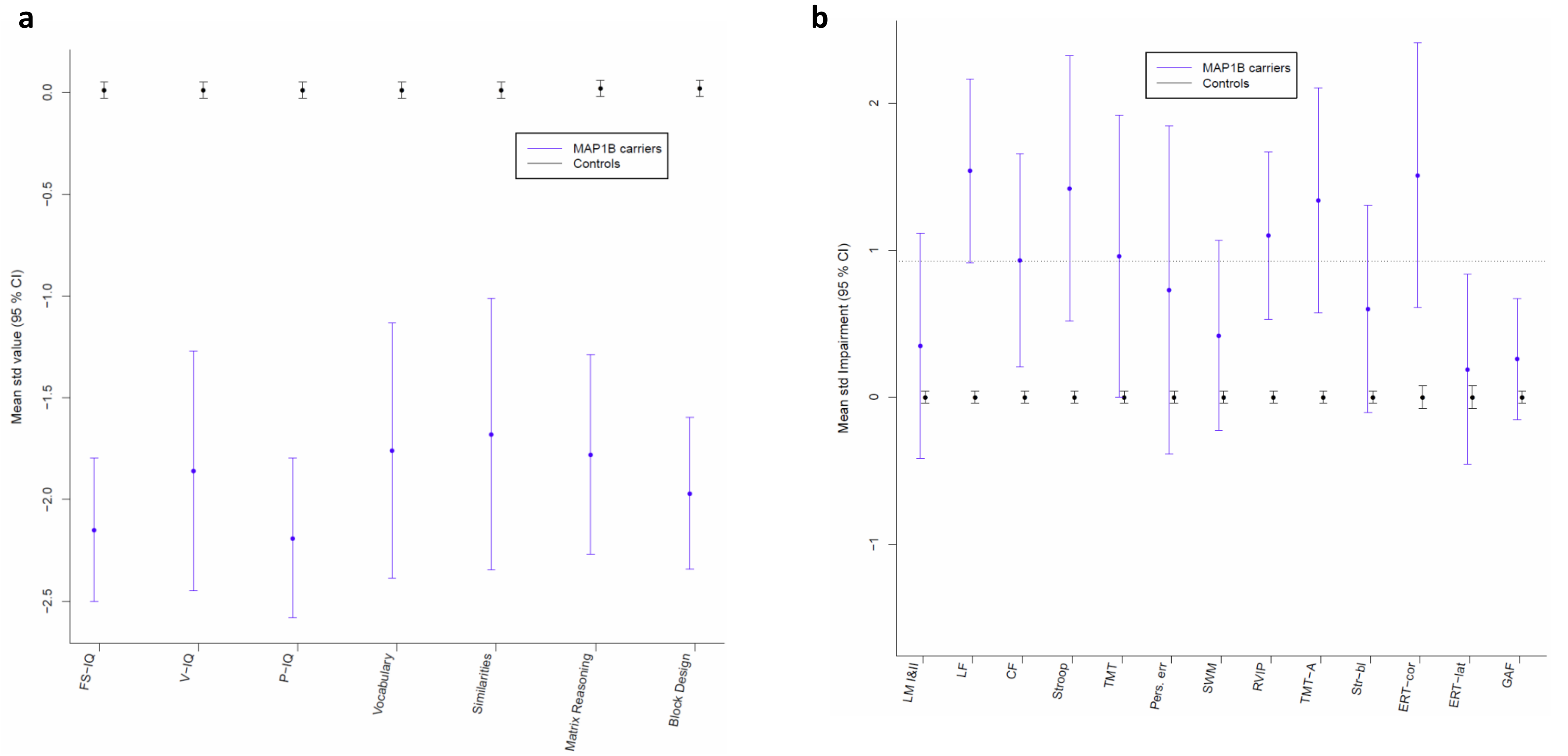

Footnote for Supplementary Figure 3. LMI&II - logical memory subtest from the Wechsler Memory Scale III (WMS-III), LF - Letter Fluency from the controlled oral word association test (COWAT), CF - Category Fluency from the category naming test, Stroop - name color minus color pad task, TMT - Trail-Making Test (refers to part B minus part A), Pers. err. - Perseverative error from the Wisconsin card-sorting test (WCST), SWM - Spatial Working Memory, RVIP - Rapid Visual Information Processing, TMT-A - Trail-Making Test part A, Str-bl - Stroop black letter, ERT-Cor and -Lat – Emotion Recognition Task percentage correct and mean response latency, GAF – Global Assessment of Functioning.

Supplementary Figure 4. Plot of IQ versus polygenic score for IQ (PGS-IQ) in *MAP1B* carriers compared with controls. The IQ score and PGS-IQ was available for 13 *MAP1B* carriers, 4 noncarrier relatives, and 2,164 control subjects. The dotted lines are the mean IQ (102.3, SD 14.7; horizontal) and mean PGS-IQ (0.03, SD 1; vertical) in the control subjects; the mean values for the *MAP1B* carriers are 68.9 (SD 10.3) and -0.2 (SD 0.6), respectively.

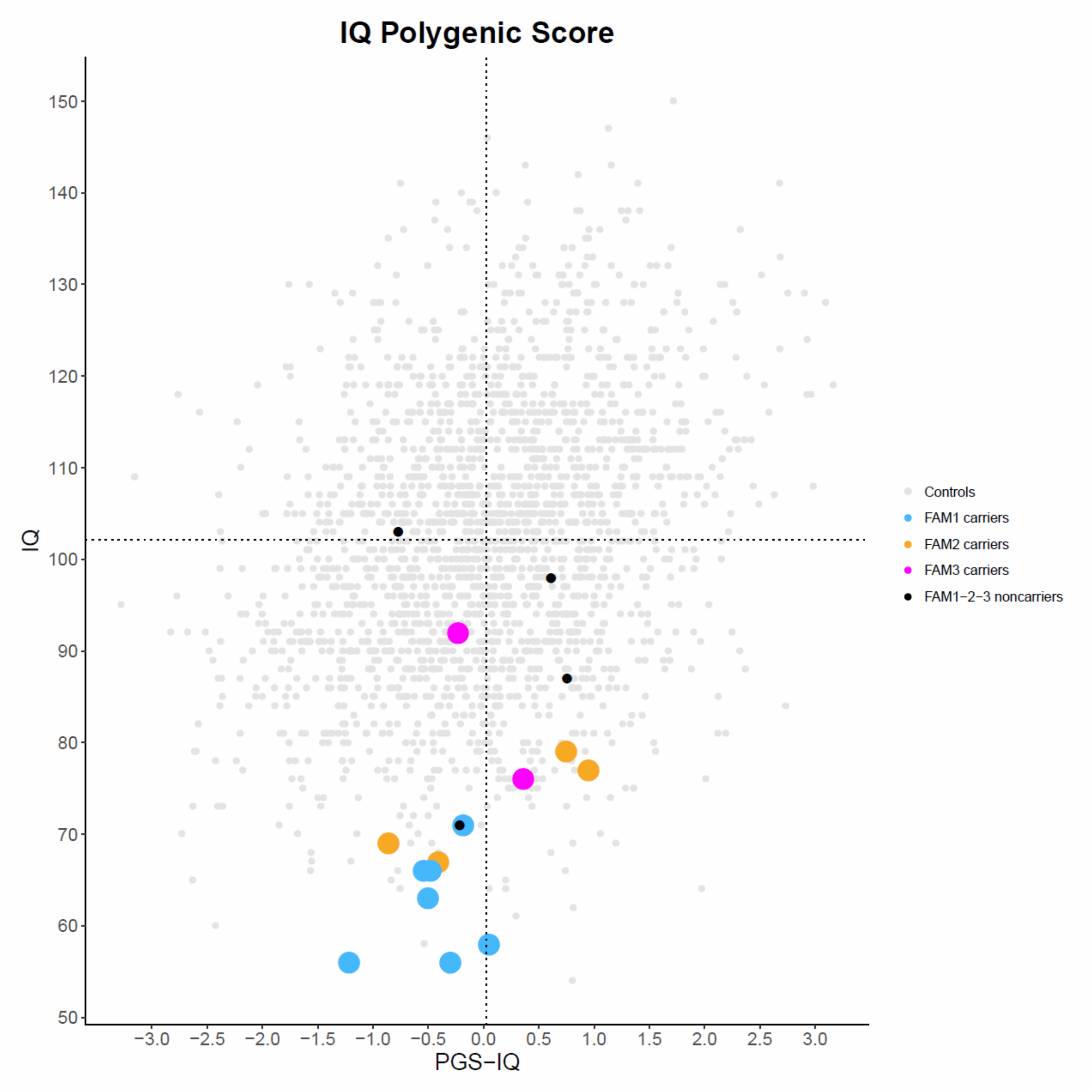

Supplementary Figure 5. Plot of IQ versus polygenic score for Educational Attainment (PGS-EDU) in *MAP1B* carriers compared with controls. The IQ score and PGS-EDU was available for 13 *MAP1B* carriers, 4 noncarrier relatives, and 2,164 control subjects. The dotted lines are the mean IQ (102.3, SD 14.7; horizontal) and mean PGS-EDU (0.03, SD 1; vertical) in the control subjects; the mean values for the *MAP1B* carriers are 68.9 (SD 10.3) and 0.09 (SD 0.93), respectively.

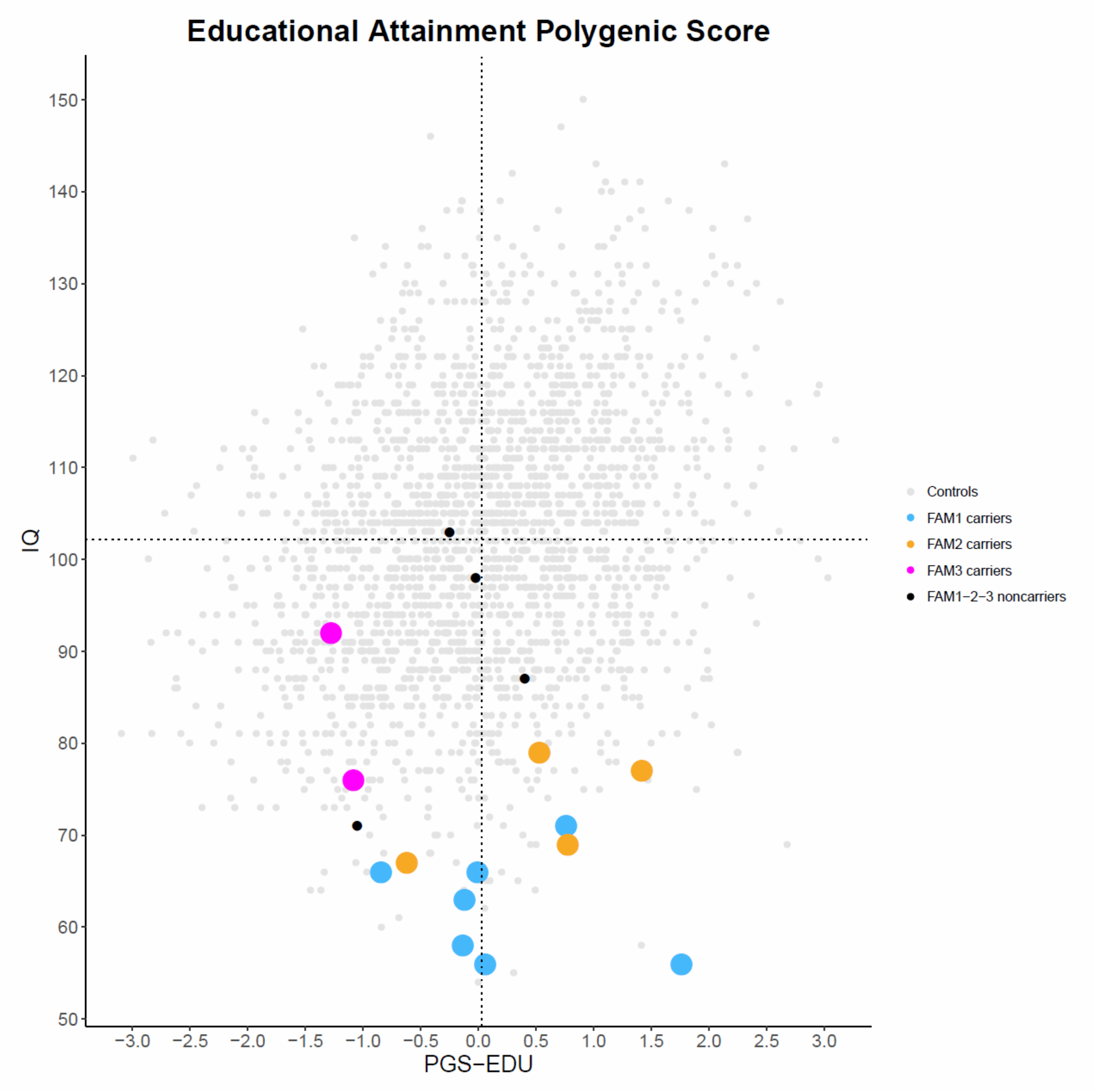

Supplementary Figure 6. a) Corpus callosum and b) brain-wide white matter volume box and whisker plots for controls (n = 856), *MAP1B* LoF carriers (n = 10; FAM1-B1,-C1,-C2,-D1,-D2,-D3, FAM2-H1,-J1,-J2, FAM3-L1), their noncarrier relatives (n = 5; FAM1-B3,-D4, FAM2-H2, FAM3-K1,-K2), noncarrier controls with low CC (β < -1.5 SD; n = 53) or low WM (β < -1.5 SD; n = 52) volume.

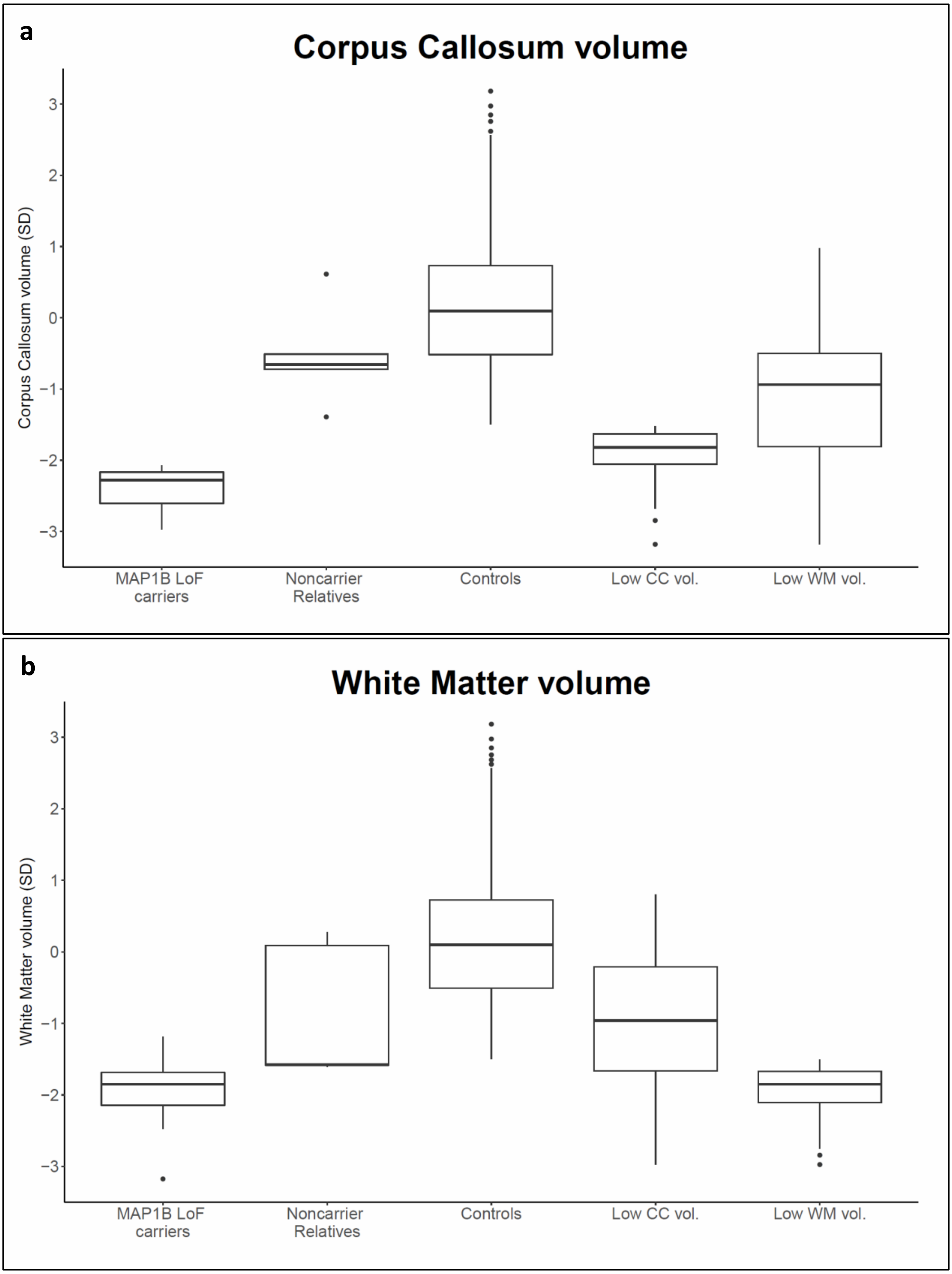

Supplementary Figure 7. Greyscale images of MAP1B antibody binding on a western blot. Western blot using the Odyssey imaging system. The greyscale images show the infrared fluorescent signals from the 700 nm channel (V5 antibody signal) and the 800 nm channel (MAP1B antibody signal) taken from the same blot. The western bands represent protein isolate from HeLa cells transfected with plasmids producing the (i) wild-type MAP1B light chain 1 (LC1), (ii) E712KfsTer10, (iii) E1032Ter and (iv) R1664Ter proteins; (v) is untransfected plasmid and (vi) is the wild-type MAP1B heavy chain (HC). For a merged image, see Figure 4.

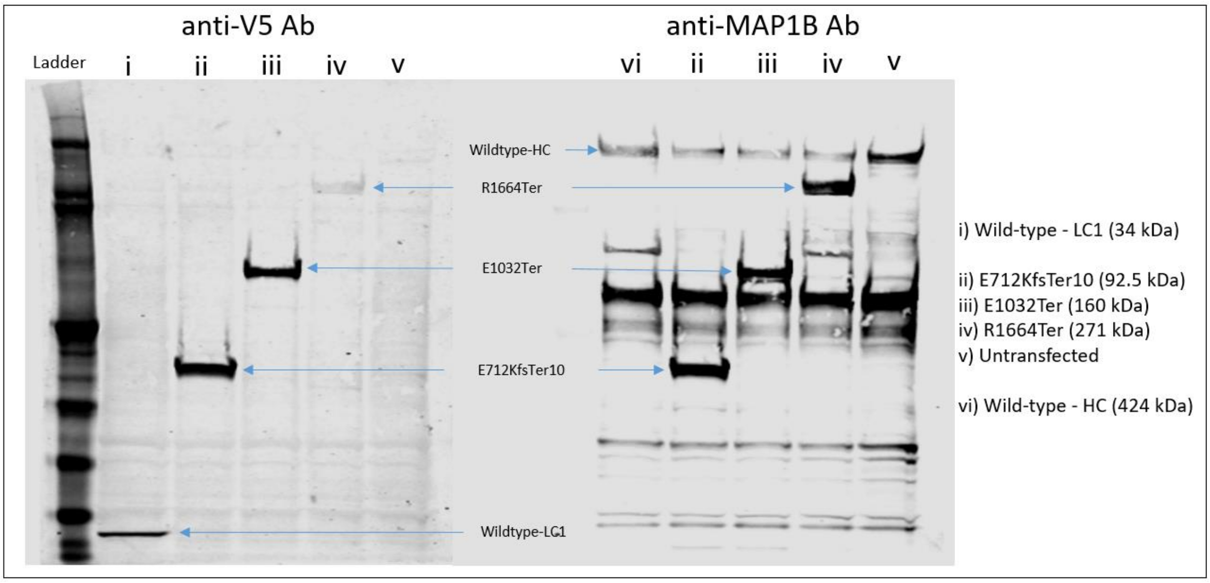

SUPPLEMENTARY REFERENCES

1. Gudmundsson E. Mat á greind fullorðinna: WASIIS (Menntamalastofnun, Reykjavik, Iceland) [The Assessment of Intelligence in Adults: WASIIS]. (2015).

2. Wechsler D. Wechsler Abbreviated Scale of Intelligence. (1999).

3. Wechsler D. *Wechsler Preschool and Primary Scale of Intelligence – Revised*. The Psychological Corporation (1989).

4. Wechsler D. *The Wechsler Intelligence Scale for Children-III*, third edition edn. The Psychological Corporation (1991).

5. Wechsler D. *The Wechsler intelligence scale for children-IV*, fourth edition edn. Pearson (2004).

6. Tripi G, Roux S, Canziani T, Bonnet Brilhault F, Barthelemy C, Canziani F. Minor physical anomalies in children with autism spectrum disorder. *Early Hum Dev* **84**, 217-223 (2008).

7. Iossifov I, *et al.* De novo gene disruptions in children on the autistic spectrum. *Neuron* **74**, 285-299 (2012).

8. Iossifov I, *et al.* The contribution of de novo coding mutations to autism spectrum disorder. *Nature* **515**, 216-221 (2014).

9. De Rubeis S, *et al.* Synaptic, transcriptional and chromatin genes disrupted in autism. *Nature* **515**, 209-215 (2014).

10. Krumm N, *et al.* Excess of rare, inherited truncating mutations in autism. *Nat Genet* **47**, 582-588 (2015).

11. Deciphering Developmental Disorders S. Prevalence and architecture of de novo mutations in developmental disorders. *Nature* **542**, 433-438 (2017).

12. Kosmicki JA, *et al.* Refining the role of de novo protein-truncating variants in neurodevelopmental disorders by using population reference samples. *Nat Genet*, (2017).

13. Firth HV, *et al.* DECIPHER: Database of Chromosomal Imbalance and Phenotype in Humans Using Ensembl Resources. *Am J Hum Genet* **84**, 524-533 (2009).

14. Liu YF, *et al.* Autism and Intellectual Disability-Associated KIRREL3 Interacts with Neuronal Proteins MAP1B and MYO16 with Potential Roles in Neurodevelopment. *PLoS One* **10**, e0123106 (2015).

15. Miller DT, *et al.* Consensus statement: chromosomal microarray is a first-tier clinical diagnostic test for individuals with developmental disabilities or congenital anomalies. *Am J Hum Genet* **86**, 749-764 (2010).

16. Korbel JO, *et al.* Paired-end mapping reveals extensive structural variation in the human genome. *Science* **318**, 420-426 (2007).

17. Pinto D, Marshall C, Feuk L, Scherer SW. Copy-number variation in control population cohorts. *Hum Mol Genet* **16 Spec No. 2**, R168-173 (2007).

18. Cooper GM, *et al.* A copy number variation morbidity map of developmental delay. *Nat Genet* **43**, 838-846 (2011).

19. Coe BP, *et al.* Refining analyses of copy number variation identifies specific genes associated with developmental delay. *Nat Genet* **46**, 1063-1071 (2014).
