# Supplementary material for: MAP1B mutations cause intellectual disability and extensive white matter deficit
Source: Nat Commun. 2018 Aug 27;9:3456. doi: 10.1038/s41467-018-05595-6 (PMC6110722; doi:10.1038/s41467-018-05595-6)
Supplement: Supplementary file 3 — Description of Additional Supplementary Files [file 41467_2018_5595_MOESM3_ESM.pdf]

Supplementary Data 1. Effects on structural MRI brain volumes, surface areas and thickness in MAP1B LoF carriers compared with controls.

List of effects on structural MRI brain volumes, surface areas and thickness in MAP1B LoF carriers compared with controls. The MAP1B-FS and Control-FS are unadjusted mean and standard deviation (SD) MRI white matter volume (mm3) and grey matter volume (mm3), surface area (mm2) and thickness (mm) values derived from FreeSurfer (FS). For each group the individuals' values were inverse normal transformed and adjusted for sex, age, age2, scanner model and ICV where appropriate. The effect (β in SD) and P-value were calculated by comparing MAP1B LoF carriers (n = 10) with controls (n = 949) using a generalised least squares regression with a variance-covariance matrix based on the kinship coefficient of each pair of individuals. Supratentorial and Brain Segment volumes (see FreeSurfer Morphometry Stats online for definitions) are presented here but were not included in Table 3 and 4. Bonferroni significance threshold was set at 0.05 / 274 = 1.8 × 10-4 (dashed line). LH, left hemisphere; RH, right hemisphere.
